# Study on effects of sweating treatment and different drying methods on the quality of *Gentianae macrophyllae* Radix

**DOI:** 10.1038/s41598-021-88511-1

**Published:** 2021-05-06

**Authors:** Qianliang Chen, Zhangyan Shi, Yedan Wang, Run Zhang, Hehe Hu

**Affiliations:** 1 College of Life Science, Northwest University, North Tabai Road, Beilin District, Xi’an, 710069 People’s Republic of China; 2 Key Laboratory of Resource Biology and Modern Biotechnology in Western China of Education Ministry, Northwest University, Xi’an, 710069 People’s Republic of China

**Keywords:** Plant breeding, Secondary metabolism, Medicinal chemistry

## Abstract

To provide basis for improving the quality of *Gentianae macrophyllae* Radix and optimising the field processing method. *Gentianae macrophyllae* Radix samples were collected from Long County. Five main active iridoids were determined by using HPLC. The HPLC fingerprints were measured and analysed by the traditional Chinese medicine chromatographic fingerprint similarity evaluation system. Water, total ash, acid insoluble ash and ethanol extract contents were determined in accordance with Chinese Pharmacopoeia (2015). Colour of the surface and powdered samples were determined by using a colorimeter. Data were analysed by SPSS11.0. Notable differences were found among samples from different sweating and drying processes, in spite of the relatively consistent overall chemical characteristics. Sweating and drying methods influenced the contents of some active ingredients and colour very significantly; moreover, they also showed significant effects on the water, ash, and ethanol extract contents. The necessity of sweating treatment in the field processing of *Gentianae macrophyllae* Radix may be insufficient. Drying in the shade and oven drying are more profitable for preserving the active constituents. *Gentianae macrophyllae* Radix should be dried directly without sweating, and drying in the shade and oven drying should be adopted preferentially to improve the quality.

## Introduction

*Gentianae macrophyllae* Radix (“Qin-jiao”), a traditional Chinese medicine, is the dried roots of the perennial herbs *Gentiana macrophylla* Pall., *G. straminea* Maxim, *G. crassicaulis Duthie ex* Burk. or *G. dahurica* Fiseh. (Gentianae). It is an important medicinal material with great market demand, and is commonly used in traditional Chinese medicine. *Gentianae macrophyllae* Radix was firstly recorded in the “Shen Nong’s herbal classic”, and eliminates wind-damp, relieves numbness and pain, dispels fever; cures arthritis, rheumatism and bone steaming hot; and treats jaundice^1^.

The quality evaluation of *Gentianae macrophyllae* Radix includes external evaluation of outside characteristics and evaluation of inherent quality. The former traditional Chinese medicine evaluation methods include evaluation of morphology, colour, odour, taste and texture. Evaluation of inherent quality is the main aspect based on chemical analysis and mainly includes the content determination of effective constituents, determination of extracts, analysis of fingerprints and checking of the contents of water, total ash, acid-insoluble ash and other items. Iridoids, which have various pharmacological activities, are the main active components of *Gentianae macrophyllae* Radix. Among them, gentiopicroside has the highest content^2,3^. In Chinese Pharmacopoeia (2015), gentiopicroside and loganin acid are the index compounds for content determination for the quality control of *Gentianae macrophyllae* Radix^1^. Therefore, content analysis of these components is important in quality evaluation, in which HPLC methods are mostly employed^4^. Some HPLC analysis of fingerprints of *Gentianae macrophyllae* Radix have been reported^5,6^. Studies have shown that many factors, such as the source of germplasm^7,8^, producing area^9^, climate and environment^10^, years of cultivation^11^ and harvesting time^12^, can influence the quality of *Gentianae macrophyllae* Radix.

Drying is an important part of processing medicinal herbs. Moreover, sweating is a special treatment used in the drying of traditional Chinese medicinal herbs. Usually, herbs are slightly sun-dried, baked or steamed, and then stacked and wrapped up to raise their temperature and make their internal water overflow, which is conducive to the subsequent drying, storage and transportation. During sweating, water congeals on the surface of the herb, making it looks like sweat^13^, and some of the chemicals in the herb change, making it change colour and smell. In the process, the change of temperature and water will change the activity of many enzymes, and then affect the transformation reaction of metabolites, leading to changes in the quality and properties of medicinal materials^14^. Proper drying methods, as well as sweating treatment, will have important effects on the quality of medicinal herbs^15^. Early studies revealed that the retention amount of gentiopicroside in *Gentianae macrophyllae* Radix which were dried after being cleaned while fresh first would be higher than that of *Gentianae macrophyllae* Radix, which were dried first before cleaning^16^. When dried at 100 °C, the loss of gentiopicroside is reduced to the minimum^17,18^. However, the effects of drying methods for gentiopicroside and other iridoids in *Gentianae macrophyllae* Radix have not been investigated. Only a few studies have investigated the sweating treatment of Chinese medicinal herbs, which mainly focused on *Magnoliae officinalis* cortex and *Ephedrae* herba. Studies on the history of field processing of *Gentianae macrophyllae* Radix show that sweating treatment of this medicinal herba appeared very late in modern times^19^. Until now, no report exists regarding the effect of sweating treatment on the quality of this crude drug. Moreover, the Chinese Pharmacopoeia^1^ continues to adopt two kinds of processing methods, namely, sweating and without sweating. In this paper, field processing of *Gentianae macrophyllae* Radix with sweating treatment and direct drying without sweating, as well as different drying methods, were carried out. The contents of five main active components and the contents of water, total ash, acid-insoluble ash and ethanol extract were determined and combined with fingerprint analysis and colour analysis to evaluate the quality of samples from different processing methods. Exploring the effects of sweating treatment and different drying methods on the quality of *Gentianae macrophyllae* Radix can favor the optimization of drying methods in field processing, and provide basis for improving the quality of this important crude drug.

## Materials and methods

### Materials and reagents

*Gentianae macrophyllae* Radix was collected from Long County cultivation base in the core authentic production region of Shaanxi Province in March 2017. They were cultivated gentiana for 4 years (3 years after transplanting) and met the standard of harvesting. Samples were identified as roots of *G. macrophyllae* Pall. by Professor Shuo-nan Wei (College of Life Science, Northwest University). Both botanical and medicinal specimens were deposited in the Department of Traditional Chinese Medicine, College of Life Science, Northwest University.

Standard substances of loganic acid (batch number: M-008-151013), 6-O-β-glucosyl-gentiopicroside (batch number: 110744-200306), swertiamain (batch number: 110744-200306) and sweroside (batch number: 110744-200306) for determination were purchased from Chengdu Ruifensi Biotechnology Co. Ltd. The standard of gentiopicroside was isolated in our laboratory and identified by using nuclear magnetic resonance spectroscopy, with a purity of more than 98%. Moreover, super pure water was purified by Milli-Q system (Millipore, Bedford, MA, USA) and filtered through a 0.22 µm filter. HPLC grade acetonitrile (TEDIA) and other reagents were of AR grade (Xi'an Chemical Reagent Factory).

### Methods

#### Preliminary processing of medicinal material herb

After excavation, non-medicinal parts were removed, leaving only the roots. Dirt on the roots was carefully brushed off with a trifle moist brush. The sample material herbs were evenly divided into seven portions, and three portions were classified into group A (sweating treatment) and three portions were classified into group B (direct drying without sweating treatment). Three kinds of drying methods (drying in shade, drying in the sun and oven drying) would be applied to dry each portion of the two groups (A and B). The remaining portion was cut into slices (2–3 mm) for quick oven drying.

##### Sweating treatment

Roots of Group A were softened by basking, and then stacked in stacks of 28 cm–22 cm–7 cm in length–width–height, covered with a woven bag, and placed at room temperature for sweating treatment. The condition of sweating was monitored closely. Sweating treatment was stopped when the roots were soft and the surface was dry.

##### Drying methods

Three methods were adopted for drying the samples Oven drying: drying in an oven at 100 °C, the model of the oven is constant temperature air-blast dryer (Shanghai Lang Gan Experimental Equipment Co., Ltd., DHG-9123A). Drying in the sun: samples were exposed in sunlight at an average temperature of 22 °C. Drying in shade: samples were placed in a cool, ventilated place to dry. When the weight of the medicinal material was constant and it was easy to be broken off, the drying was completed. Processes of samples from group A and B corresponded to the same drying method to maintain the same drying time.

#### Determination of main active constituents

##### Apparatus and chromatograph condition

Agilent 1200 high performance liquid chromatography (Agilent), photodiode array detector, automatic injector and column temperature box.

**Figure 1:**
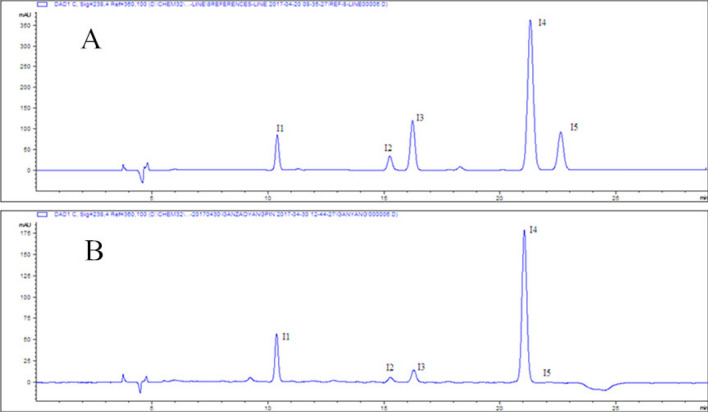
HPLC Chromatograms of standards and sample. (**A**) Standard of five iridoid glucosides, (**B**) sample.

The chromatographic conditions based on the method in the literature^20^ were slightly modified. AE.LICHROM-C_18_ chromatographic column (250 mm × 4.6 mm, 5 μm, Lanzhou Zhongke Analytical Technology Co., Ltd.). The mobile phase: acetonitrile (A)-0.2% acetic acid (B), gradient elution, 0–8 min, 9%-11% (A); 8–16 min, 11–12% (A); 16–19 min, 12% (A); 19–20 min, 12–9% (A); 20–29 min, 9% (A); the flow rate: 0.8 mL/min. Column temperature was 30 ℃. Detection wavelength: 238 nm. The chromatograph under these conditions is shown in Fig. 1.

##### Preparation of standard solution

Reference samples of the five iridoid glucosides were accurately weighed and put in a 5-mL volumetric flask and dissolved in 30% methanol to form standard stock solutions. Appropriate volumes of stock solutions were mixed and diluted to form a mixed standard solution, in which the concentrations of loganic acid (I1), 6-O-β-glucosyl-gentiopicroside (I2), swertiamarin (I3), gentiopicroside (I4) and sweroside (I5) were 0.0334, 0.0332, 0.0574, 0.2840 and 0.0606 mg/mL, respectively.

##### Preparation of test solution

The dried samples were crushed and screened over a No. 3 sieve. About 500 mg powder was accurately weighed and extracted with about 20 mL of methanol by ultrasonication (100 W, 40 kHz) for 30 min in a 25-mL volumetric flask. The extract was cooled to room temperature, adjusted to the volume with methanol, mixed well and filtered through a 0.22 µm filter membrane.

#### Method validation and wavelength selection of determination

The calibration curves were constructed by injecting the standard solution. Plot of the peak area versus analyte quantity resulted in calibration equations of Y = 758.22 X + 0.3173 (r = 1) for I1; Y = 909.68X-2.4638 (r = 1) for I2; Y = 1081.9X − 4.1212 (r = 1) for I3; Y = 1443.8X − 5.0511 (r = 1) for I4; and Y = 1585.4X − 12.688 (r = 0.9999) for I5, indicating that the linear relationship of the determination was good.

To assess the precision of the method, the injected standard solutions were used. The intra- and inter-day coefficients of variation were both less than 5.0% (n = 5), which were satisfactory. The stability and recovery were evaluated according to the Chinese Pharmacopoeia^1^.

Chromatograms at 214, 238, 254 and 272 nm were measured. At the wavelength of 238 nm, five iridoid glucosides showed relatively large absorption, and their quantitative accuracy values were higher; therefore, 238 nm was chosen as the detection wavelength for determination.

#### Fingerprint analysis

**Figure 2:**
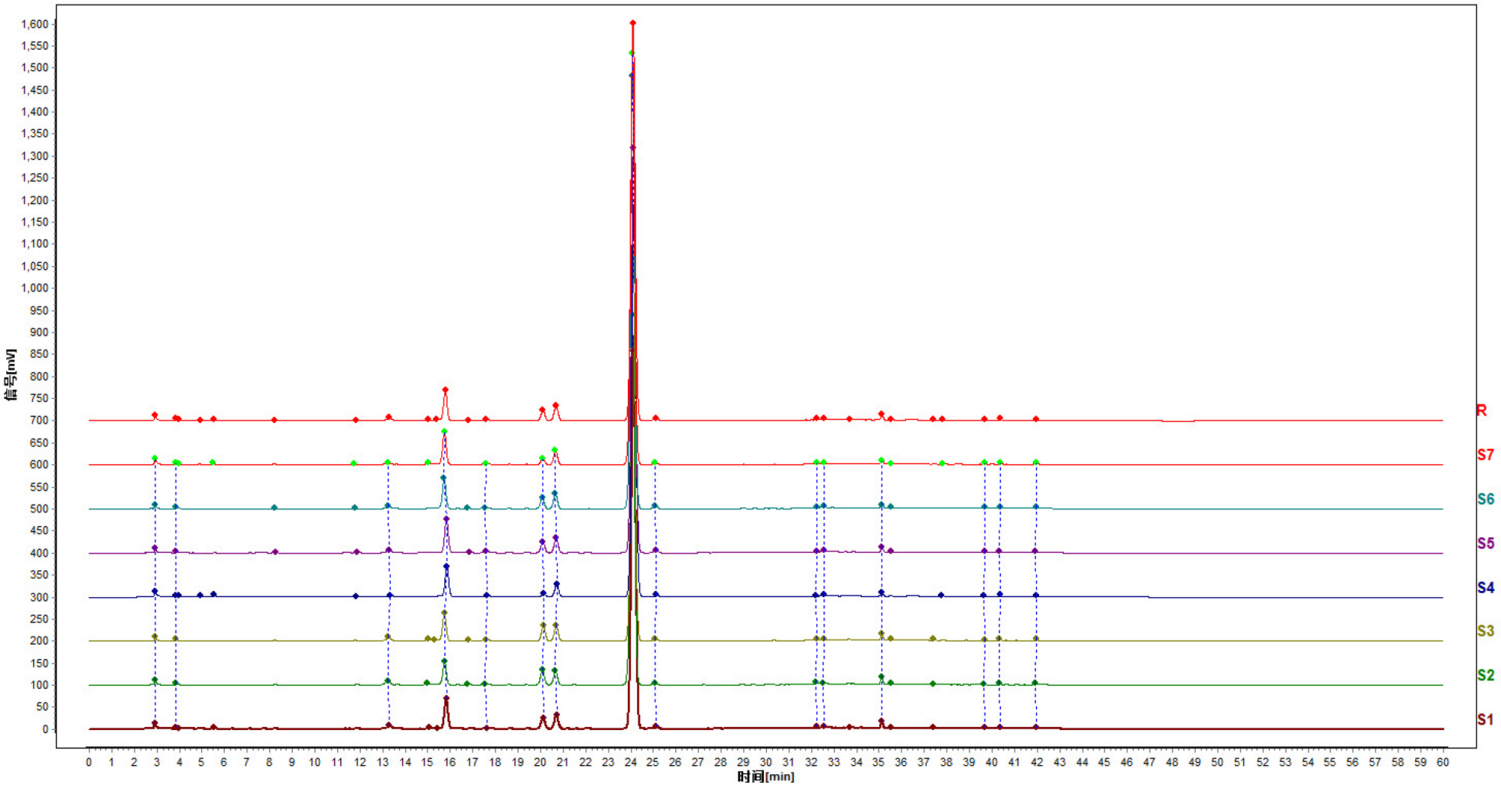
Fingerprint of *Gentianae macrophylla* radix samples with different sweating and drying treatment. *R* reference fingerprint, *S1–S7* fingerprint of each sample.

The fingerprint analysis was carried out according to the method in the literature^11^. Typical fingerprints are shown in Fig. 2.

#### Determination of water, ash and ethanol extract

The moisture content was measured by loss on drying method, using glass evaporating dish (100 mm, Beijing Glass Co., Ltd). Ash content was determined by residue on ignition method and muffle furnace (2001 model, TDW series temperature controller, Wenzhou Shuangyu Instrument Factory) was used. The ethanol soluble substance was extracted by Soxhlet extraction, using Soxhlet extractor (250 mL, Tianjin Glass Instrument Factory). These determinations were carried out following the standards for Gentianae macrophyllae Radix in Chinese Pharmacopoeia^1^: the second method of general rule 0832 for water; the general rule 2302 for total ash and acid-insoluble ash; the hot-maceration method of the general rule 2201 for ethanol soluble extract.

#### Colour analysis

##### Apparatus and determination condition

NS800 colorimeter (Shenzhen 3nh Technology Co., Ltd.). Light source D65, LED blue light excitation; measurement geometry 8°/day; silicon photodiode detector; measurement aperture 4 mm; location mode: lighting location and cross positioning. Colour space CIE, L*a*b*, among them, L* is the brightness of the colour, L* = 0 is black, L* = 100 is white; a* is between red and green, a* negative value is green, positive value is red; b* is between yellow and blue, b* negative value is blue, positive value is yellow.

##### Colour determination of surface and powder

Both surface colour of *Gentianae macrophyllae* Radix and powder were determined. A total of three roots were randomly selected for each sample for determination. Because the bark colour of same root may vary, to ensure accuracy and consistency, the colours of the three parts, namely, the top part, medium part and lower part, of one same root were determined; moreover, the measurement of each part was repeated thrice, with the RSD < 3%. The powder was uniform in colour; thus, each sample was determined thrice.

#### Data analysis

All the data were input into EXCEL software and analysed by SPSS11.0. The traditional Chinese medicine chromatographic fingerprint similarity evaluation system version 2.0 (National Pharmacopoeia Committee 2012) was adopted in the fingerprint analysis.

### Statement

The plant materials used in this experiment were cultivated Gentiana macrophylla, purchased from Badu Township, Longxian County. Local planting of *Gentiana macrophylla* complies with PRC and local laws and regulations. The experimental material was planted *Gentiana macrophylla*, which is not a protected plant. It is a cash crop planted in a traditional cultivation method and does not require specific permission for harvest.

## Results

### Results of sweating treatment and drying

Sweating treatment of *Gentianae macrophyllae* Radix lasted two weeks. During the course, the temperature in the crude drug stacks increased. The medicinal material shrank, the surface felt moist and the colours darkened. The surface colour of the medicinal material that was dried after sweating was darker than that of the directly dried samples without sweating.

Different drying methods took different durations. Slice oven drying was the fastest drying method and took about 3.5 h. The time of slice drying was used as a reference to determine the drying time of other drying methods. Experimental results showed that oven drying had the shortest drying time among the three methods and can be completed in a few hours. Drying in the shade took the longest time of 2 weeks. Sun-drying took the median amount of time. Under the conditions of our experiment, with the average daily sun of about 7 h, drying *Gentianae macrophyllae* Radix can be finished in a week. The specific drying time is shown in Table 1.

**Table 1:** The drying time of medicinal materials.

Sample	Oven drying after sweating treatment	Oven drying directly	Drying in the sun after sweating treatment	Drying in the sun directly	Drying in shade after sweating treatment	Drying in shade directly	Slice oven drying
Drying time (h)	3.5	3.5	51.9	51.9	384.0	384.0	3.5

### Contents of main active constituents

The results of the determination of five components in each sample are shown in Table 2. Double-factor variance analysis of the sweating treatment and drying method showed that the loganic acid (I1) in the samples that were dried in the sun and dried in the shade after sweating treatment were significantly lower than those in the other samples. Moreover, the content of 6-O-β-glucosyl-gentiopicroside (I2) in the oven-dried samples without sweating and directly slice-dried samples were significantly lower than those in the other samples. Additionally, the content of gentiopicroside (I4) in the sample dried in the shade directly without sweating was significantly higher than those in the other samples. No significant difference in the contents of swertiamarin (I3) and sweroside (I5) were found in the samples. Furthermore, the sum of the contents of the five active constituents in the samples dried in the shade without sweating was significantly higher than those in the other samples; whereas, the sum was significantly lower in the sun-dried samples with sweating.

**Table 2:** The contents of five iridoids in each sample (M ± S, n = 3).

Sample	I1 (%)	I2 (%)	I3 (%)	I4 (%)	I5 (%)	Sum of the five iridoids (%)
Oven drying after sweating treatment	1.139 ± 0.017	0.395 ± 0.000	0.386 ± 0.004	7.669 ± 0.136	0.051 ± 0.003	9.641 ± 0.117
Oven drying directly	1.171 ± 0.020	0.111 ± 0.006**	0.349 ± 0.016	8.092 ± 0.049	0.035 ± 0.001	9.757 ± 0.093
Drying in the sun after sweating treatment	0.825 ± 0.008**	0.442 ± 0.007	0.380 ± 0.020	7.357 ± 0.027	0.043 ± 0.001	9.047 ± 0.047**
Drying in the sun directly	1.182 ± 0.023	0.369 ± 0.005	0.383 ± 0.008	8.000 ± 0.100	0.053 ± 0.003	9.961 ± 0.099
Drying in shade after sweating treatment	0.996 ± 0.019**	0.433 ± 0.002	0.427 ± 0.004	8.132 ± 0.008	0.049 ± 0.003	10.037 ± 0.032
Drying in shade directly	1.180 ± 0.032	0.374 ± 0.014	0.400 ± 0.001	8.766 ± 0.085**	0.048 ± 0.003	10.768 ± 0.101**
Slice oven drying	1.209 ± 0.005	0.194 ± 0.001**	0.371 ± 0.012	8.129 ± 0.163	0.041 ± 0.009	9.946 ± 0.160

I1 Loganic acid, I2 6-O-β-glucosyl-gentiopicroside, I3 Swertiamarin, I4 Gentiopicroside, I5 Sweroside (*P < 0.05, **P < 0.01, calculate by SPSS).

The results of ANOVA (Table 3) showed that sweating treatment had a significant influence on the contents of I1, I2 and I4. The contents of I1 and I4 and the sum of the contents of the five active constituents in the samples with sweating treatment were significantly lower than the direct drying samples, whereas I2 in the samples with sweating treatment was significantly higher than the direct drying samples. The contents of I3 and I5 did not differ significantly between the sweating and non-sweating samples.

**Table 3:** The effect of sweating treatment on contents of five iridoids.

Factor	I1 (%)	I2 (%)	I3 (%)	I4 (%)	I5 (%)	Sum of the five iridoids (%)
Sweating treatment	0.987 ± 0.141**	0.424 ± 0.022**	0.398 ± 0.025	7.719 ± 0.354**	0.048 ± 0.004	9.575 ± 0.450**
Without sweating treatment	1.178 ± 0.021	0.285 ± 0.135**	0.378 ± 0.025	8.286 ± 0.379	0.045 ± 0.008	10.162 ± 0.484

I1 Loganic acid, I2 6-O-β-glucosyl-gentiopicroside, I3 Swertiamarin, I4 Gentiopicroside, I5 Sweroside (*P < 0.05, **P < 0.01, calculate by SPSS).

The results of ANOVA for the different drying methods are shown in Table 4. The content of I4 and the sum of the five active constituents in the samples dried in shade were extremely significantly higher than in the samples from other drying methods, I3 was significantly higher than in the samples from other drying methods. In oven-dried samples, I1 was significantly higher than those in the other samples, whereas I2 was significantly lower. The contents of I1 and I4 and the sum of the five active constituents in the sun-dried samples were lowest among all samples.

**Table 4:** The effect of drying methods on contents of five iridoids.

Factor	I1 (%)	I2 (%)	I3 (%)	I4 (%)	I5 (%)	Sum of the five iridoids (%)
Oven dying	1.155 ± 0.024**	0.253 ± 0.164**	0.368 ± 0.024	7.881 ± 0.258**	0.043 ± 0.010	9.699 ± 0.109 **
Drying in the sun	1.004 ± 0.207**	0.405 ± 0.043	0.382 ± 0.013	7.678 ± 0.376**	0.048 ± 0.006	9.504 ± 0.532**
Drying in shade	1.088 ± 0.108**	0.404 ± 0.035	0.414 ± 0.016*	8.449 ± 0.369**	0.049 ± 0.003	10.403 ± 0.426**

I1 Loganic acid, I2 6-O-β-glucosyl-gentiopicroside, I3 Swertiamarin, I4 Gentiopicroside, I5 Sweroside (*P < 0.05, **P < 0.01, calculate by SPSS).

### Results of fingerprint analysis

The fingerprint data collected by the chromatograph was imported into the chromatographic fingerprint evaluation software of similarity analysis (National Pharmacopoeia Committee 2012). After automatic matching and multi-point correction, a reference chromatograph was generated, as shown in Fig. 2. A total of 26 peaks were present in the chromatograph, among which 15 peaks were common peaks. These 15 peaks can fully represent the whole chromatograph, because their total area accounted for 99% of the total fingerprint area. The similarity calculation showed that the similarity of all samples was above 0.999, indicating that the holistic chemical characteristics of each sample with different sweating treatments and drying were relatively alike and only slightly differentiated.

### Results of determination of water, ash and ethanol extract

The results of determination of water, ash and ethanol extract contents are shown in Table 5. All samples reached the prescribed standard of *Gentianae macrophyllae* Radix in Chinese Pharmacopoeia (2015 edition). Two-factor analysis of variance showed no significant difference among the water, ash and ethanol extract contents of each sample.

**Table 5:** Determination of water, total ash, acid-insoluble ash and ethanol extracts (M ± S, n = 3).

Sample	Water (%)	Total ash (%)	Acid insoluble ash (%)	Ethanol extracts (%)
Oven drying after sweating treatment	6.531 ± 0.047	6.210 ± 1.296	1.349 ± 1.578	38.755 ± 1.241
Oven drying directly	7.617 ± 0.016	7.196 ± 0.045	2.669 ± 0.094	38.760 ± 0.813
Drying in the sun after sweating treatment	7.780 ± 0.266	7.064 ± 0.192	3.015 ± 0.776	38.875 ± 0.177
Drying in the sun directly	6.368 ± 0.051	6.836 ± 0.118	2.878 ± 0.447	40.997 ± 0.546
Drying in shade after sweating treatment	7.984 ± 0.039	8.722 ± 0.012	2.563 ± 0.091	43.783 ± 0.350
Drying in shade directly	7.461 ± 0.086	7.257 ± 0.417	2.630 ± 0.045	41.930 ± 0.223
Slice oven drying	6.182 ± 0.023	7.647 ± 0.076	2.763 ± 0.186	40.258 ± 0.499

Single factor analysis (Table 6) showed that sweating treatment had a significant effect on ethanol extract content. The ethanol extract content in the non-sweating samples was higher than that in the sweating samples. The differences among water, total ash and acid-insoluble ash contents were not significant.

**Table 6:** The effect of sweating treatment on water, total ash, acid-insoluble ash and ethanol extracts.

Factor	Water (%)	Total ash (%)	Acid insoluble ash (%)	Ethanol extracts (%)
Sweating treatment	7.309 ± 0.619	7.332 ± 1.284	2.309 ± 1.102	38.804 ± 0.670
Without sweating treatment	7.271 ± 0.739	7.097 ± 0.282	2.735 ± 0.235	42.237 ± 1.305*

(*P < 0.05, **P < 0.01, calculate by SPSS).

The results of ANOVA for the different drying methods are shown in Table 7. Water content in the oven-dried samples was significantly lower than those in the sun-dried and dried in the shade. Total ash of the sun-dried samples was significantly higher than those of the oven-dried and dried in shade. No significant differences were found in the acid-insoluble ash and ethanol extract contents among the three drying methods.

**Table 7:** The effect of drying methods on water, total ash, acid-insoluble ash and ethanol extracts.

Factor	Water (%)	Total ash (%)	Acid insoluble ash (%)	Ethanol extracts (%)
Oven dying	6.360 ± 0.160**	7.027 ± 0.885	2.270 ± 1.008	40.010 ± 1.201
Drying in the sun	7.800 ± 0.213	7.989 ± 0.879**	2.596 ± 0.070	41.272 ± 2.945
Drying in shade	7.621 ± 0.245	6.950 ± 0.185	2.947 ± 0.523	40.402 ± 1.771

(*P < 0.05, **P < 0.01, calculate by SPSS).

### Results of colour determination

**Figure 3:**
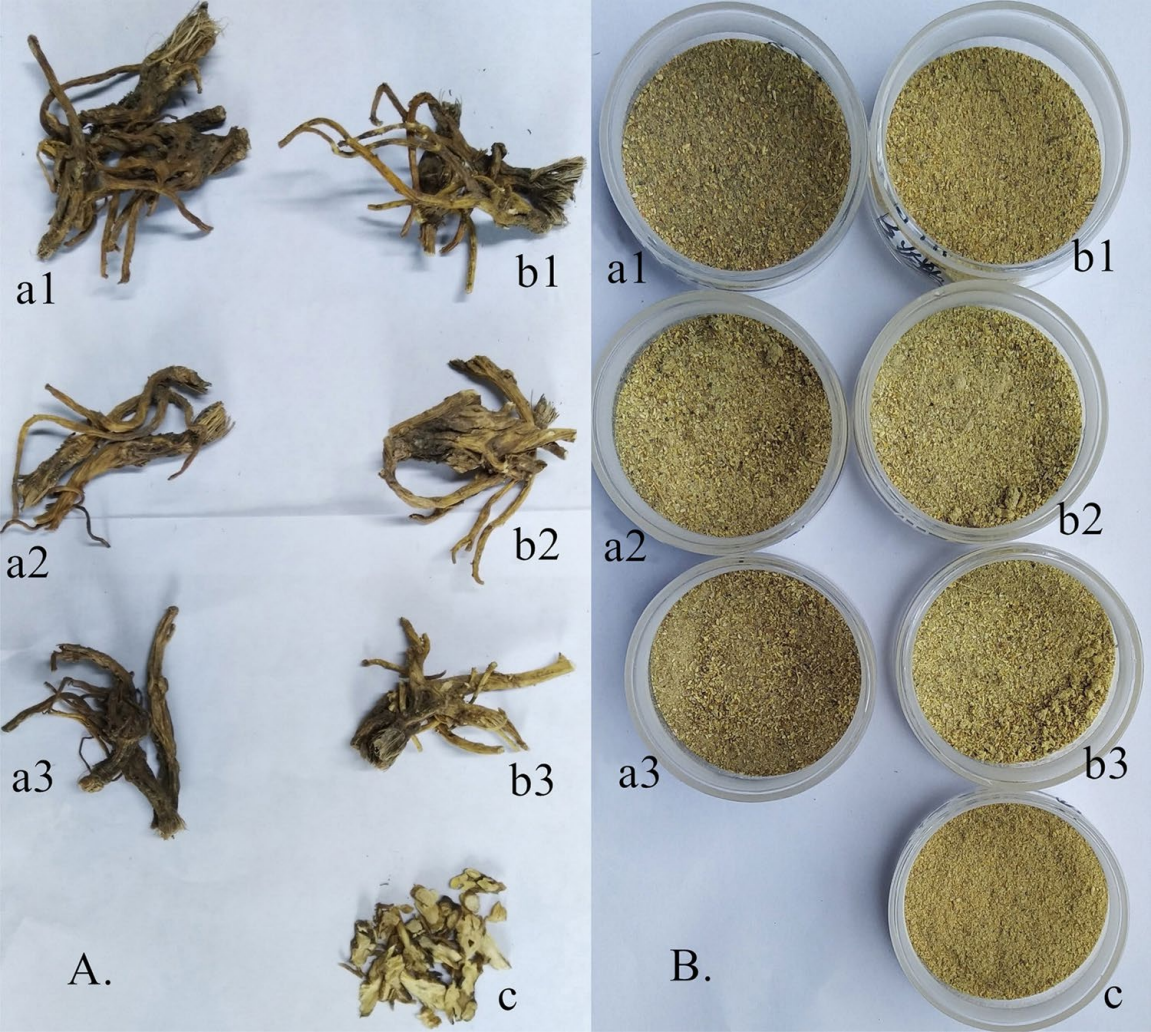
Appearance of *Gentianae macrophyllae* radix with different processing and their powder. (**A**) *Gentianae macrophyllae* radix with different processing, (**B**) Powder. a1 Oven drying after sweating treatment; a2 Drying in the sun after sweating treatment; a3 Drying in shade after sweating treatment; b1 Oven drying directly; b2 Drying in the sun directly; b3 Drying in shade directly; c Slice oven drying.

As shown in Fig. 3, distinct colour differences among samples from different processing methods can be seen by naked eye, either at the surface colour or drug powder colour. The results of colour determination are shown in Table 8. The results of ANOVA according to sweating treatment or non-sweating treatment and different drying methods are shown in Tables 9 and 10, respectively. The L* and b* values of surface colour of the samples from sweating treatment were extremely significantly lower, and the a* value of the sweating sampled was significantly lower than the non-sweating samples. Meanwhile, the L* value of powder colour of samples with sweating treatment was significantly lower than the non-sweating samples. This revealed that sweating can notably deepen surface colour. However, Table 10 revealed that only the a* value of surface colour of the sun-dried sample was significantly higher than those of samples from other drying methods. Moreover, a* and b* values of powder colour of the oven-dried samples were significantly higher than those in the samples from shade-dried and sun-dried methods.

**Table 8:** Determination of color (M ± S, n = 3).

Sample	Surface			Powder		
	L*	a*	b*	L*	a*	b*
Oven drying after sweating treatment	43.376 ± 1.785	6.304 ± 1.322	15.334 ± 0.384	61.377 ± 0.012	5.210 ± 0.000	20.823 ± 0.025
Oven drying directly	54.291 ± 0.754	6.910 ± 0.134	21.645 ± 2.100	65.420 ± 0.182	5.160 ± 0.217	20.657 ± 0.205
Drying in the sun after sweating treatment	48.684 ± 7.087	6.379 ± 0.792	19.003 ± 2.255	64.790 ± 0.087	4.297 ± 0.006	20.517 ± 0.021
Drying in the sun directly	55.670 ± 1.128	7.814 ± 0.090	23.272 ± 0.411	69.993 ± 0.006	3.7867 ± 0.006	19.717 ± 0.015
Drying in shade after sweating treatment	38.655 ± 1.913	5.397 ± 0.308	15.378 ± 2.720	61.353 ± 0.006	4.530 ± 0.000	20.037 ± 0.015
Drying in shade directly	53.409 ± 1.421	6.083 ± 0.058	20.557 ± 0.537	69.120 ± 0.026	3.660 ± 0.017	20.103 ± 0.006
Slice oven drying	–	–	–	66.367 ± 0.021	4.660 ± 0.026	20.720 ± 0.030

**Table 9:** The effect of sweating treatment on color.

Factor	Surface			Powder		
	L*	a*	b*	L*	a*	b*
Sweating treatment	43.572 ± 5.758^2)^	6.027 ± 0.917^1)^	16.572 ± 2.805^2)^	62.507 ± 1.713^2)^	4.679 ± 0.411	20.459 ± 0.344
Without sweating treatment	54.457 ± 1.392	6.935 ± 0.756	21.825 ± 1.618	68.178 ± 2.105	4.202 ± 0.729	20.159 ± 0.422

(^1)^P < 0.05, ^2)^P < 0.01, calculate by SPSS).

**Table 10:** The effect of drying methods on color.

Factor	Surface			Powder		
	L*	a*	b*	L*	a*	b*
Oven dying	48.834 ± 6.103	6.607 ± 0.904	18.490 ± 3.998	63.398 ± 2.218	5.185 ± 0.140^2)^	20.740 ± 0.159^2)^
Drying in the sun	52.177 ± 5.936	7.097 ± 0.933^1)^	21.137 ± 2.751	67.392 ± 2.850	4.042 ± 0.279	20.117 ± 0.438
Drying in shade	46.032 ± 8.220	5.740 ± 0.425	17.967 ± 3.335	65.237 ± 4.254	4.095 ± 0.477	20.070 ± 0.038

(^1)^P < 0.05, ^2)^P < 0.01, calculate by SPSS).

## Discussion

### Effect of sweating treatment

The gentiopicroside and loganic acid are important contents of *Gentianae macrophyllae* Radix quality evaluation in Chinese Pharmacopoeia. Therefore, it is an important aspect to investigate the influence of sweating treatment on the main components of iridoid glycosides. In this paper, the results showed that the contents of main active iridoid glycosides in *Gentianae macrophyllae* Radix treated by sweating and that treated without sweating were significantly different. The contents of I1 and I4, the two index components in Chinese Pharmacopoeia, in non-sweating herbs were significantly higher than those in the sweating herbs. The 6-O-β-glucosyl-gentiopicroside (I2) in the sweat samples was significantly higher than that in the non-sweat samples, but its content was relatively low, which had little effect on the overall quality condition. The above results are consistent with the previous research results^21,22^. The content of ethanol extract of *Gentianae macrophyllae* Radix with sweating treatment was significantly lower than without sweating treatment. This may be due to the fact that during sweating, the cells of root tissue remain breathing for a period of time, consuming more substances than the samples dried directly without sweating, resulting in a decrease in extract content.

### Effect of different drying methods

Studies have shown that the drying method will affect the content of medicinal constituents. Some components of medicinal materials in the drying process will increase and some may decompose. These effects would be highly remarkable for crude drugs in which the main components are compounds with active chemical properties. When drying *Lonicerae japonicae* flos at low temperature, the content of secoxyloganin, an iridoid glycoside, increases, whereas the content of morroniside tends to reduce^23^. Under different drying conditions, the trends of composition changes differ. The retention of each component is different, and results in the difference of the internal quality of medicinal materials. Similar to secoxyloganin and morroniside in *Lonicerae japonicae* flos, the main effective components of *Gentianae macrophyllae* Radix, such as gentiopicrside and loganin acid, are iridoid glycosides with active chemical properties. These would be affected by drying methods in all probability during field processing. Based on comprehensively analysis of the contents of five main components, drying in shade was the most conducive method for preserving the effective components, in spite of I1 content being lower. The total contents of main constituents in oven-dried samples were lower than those in shade-dried samples; however, oven drying had the advantage of faster drying time. The oven-drying method had two advantages to drying in the sun: the contents of the two index components (I1 and I4) in the pharmacopoeia were high; the time consumption was short. Therefore, drying in shade was the best method, oven drying was the second, and drying in the sun was the least effective for retaining the active components. This may be because prolonged heat during drying in the sun destroys the active ingredients^22^. The water contents of all samples from three drying methods were all less than 8%, reaching the prescribed standard of China Pharmacopoeia (2015 edition). Water content in the oven-dried sample was significantly lower than those in the other samples. Moreover, the total ash and acid-insoluble ash contents of all samples reached the prescribed standard of China Pharmacopoeia. Among them, the acid-ash content of the sample dried in shade was highest among all samples, which may be due to the fact that the samples were easily polluted by environmental dust during the long drying in the shade. Therefore, the environment must be kept clean when herbs are being dried in the shade.

### Effect of sweating treatment and drying methods on colour

Colour is an important aspect of quality evaluation of traditional Chinese medicinal materials. Traditionally, the faint yellow variety of *Gentianae macrophyllae* Radix is the best^24^. This means the lighter colour represents relatively good quality. Sweating treatment significantly affected the colour of *Gentianae macrophyllae* Radix, especially the surface colour, and can notably darken the colour of medicinal materials. This is consistent with the research results in the literature^21^. The reason for this phenomenon may be the activation of some enzymes during sweating, which causes the hydrolysis and repolymerization of some iridoid glycosides to form dark precipitation. Therefore, sweating treatment is not good for the appearance of *Gentianae macrophyllae* Radix. The effect of drying method on colour is less apparent than that of sweating; however, drying in the sun can redden the surface of the medicinal materials, and oven drying can make the colour of the medicinal powder more red and yellow.

## Conclusion

Fingerprint analysis showed that the overall chemical characteristics of *Gentianae macrophyllae* Radix in different treatments were relatively consistent, and sweating treatment and drying methods would not essentially differentiate the overall quality of this crude drug. However, some remarkable differences were found in the quality of dried herbs under different sweating and drying conditions. In terms of the contents of active constituents, non-sweating *Gentianae macrophyllae* Radix was superior to those that underwent sweating treatment. In addition, no record of sweating treatment of *Gentianae macrophyllae* Radix exists in ancient Materia Medica. Moreover, sweating can significantly darken the surface colour, reducing the appearance quality. And sweating treatment often takes weeks longer as well as it only appeared in the field processing of *Gentianae macrophyllae* Radix very late in modern time. Therefore, the basis for sweating treatment of *Gentianae macrophyllae* Radix may be insufficient. Among these three ways of drying: oven drying, drying in the sun and drying in shade, drying in shade and oven drying were more conducive to preserving the active constituents. The quality of medicinal materials dried by these two methods was better. In summary, in the field processing of *Gentianae macrophyllae* Radix, herbs should be dried directly and without sweating, and drying in the shade or oven drying should be adopted preferentially to improve the quality.
